# The Role of ADAM9 and MMP9 in Diabetic Retinopathy: Insights from Ocular Parameters

**DOI:** 10.3390/ijms26178436

**Published:** 2025-08-29

**Authors:** Mehmet Ali Gul, Duygu Tozcu Yilmaz, Nihat Aydin, Melek Tufek, Mustafa Capraz

**Affiliations:** 1 Department of Medical Biochemistry, Faculty of Medicine, Amasya University, Amasya 05100, Türkiye; 2 Department of Physiology, Faculty of Medicine, Amasya University, Amasya 05100, Türkiye; duygu.tozcu@amasya.edu.tr; 3 Department of Ophthalmology, Amasya University, Sabuncuoglu Serafeddin Training and Research Hospital, Amasya 05100, Türkiye; nihat.aydin@amasya.edu.tr (N.A.); melek.tufek@amasya.edu.tr (M.T.); 4 Department of Internal Medicine, Amasya University, Sabuncuoglu Serafeddin Training and Research Hospital, Amasya 05100, Türkiye; m.capraz@amasya.edu.tr

**Keywords:** ADAM9, MMP9, diabetic retinopathy

## Abstract

The focus in this study was to investigate the proteolytic functions of matrix metalloproteinases (MMPs) and a disintegrin and metalloprotease (ADAM) proteins in the progression of diabetic retinopathy (DR) and to evaluate their potential as therapeutic targets for eye diseases. This study involved three groups: non-proliferative diabetic retinopathy patients (NPDR) (n = 27), proliferative diabetic retinopathy patients (PDR) (n = 32), and a control group of 30 healthy individuals. Serum levels of ADAM9 and MMP9 were measured across these groups for comparative analysis. Serum ADAM9 levels were significantly lower in the NPDR and PDR groups than in the control group (*p* = 0.031, *p* < 0.001). Although ADAM9 levels were lower in the PDR group than in the NPDR group, this difference was not significant (*p* = 0.142). Serum MMP9 levels in the PDR group were significantly lower than those in both the control and NPDR groups (*p* = 0.039, *p* = 0.013). The findings of this study indicate that ADAM9, MMP9, and left-eye ocular parameters may have potential value in the assessment of DR. The notable variation in the MMP9 marker in the proliferative stage, as opposed to its stability in the non-proliferative stage, suggests a distinct role in retinopathy staging. This underscores the specific importance of MMP9 in the proliferative stage.

## 1. Introduction

The incidence of diabetes continues to increase, affecting millions of people. Diabetic retinopathy (DR) is one of the most feared complications of diabetes. It is a slowly progressive complication arising from degenerative changes in retinal blood vessels. In the initial stages of DR, the disease may be asymptomatic; however, if not treated, it can eventually cause blindness [1,2,3]. Sustained hyperglycemia is a primary contributor to the onset of DR; however, the molecular mechanisms underlying glucose-induced retinal injury remain incompletely understood. The advancement of the condition correlates with enhanced apoptotic activity affecting retinal vascular endothelial cells, Müller glial cells, and retinal ganglion neurons [4]. Recent studies suggest that control of circulating blood glucose and blood pressure has the potential to delay the progression of retinopathy in diabetic patients, but that maintaining normal blood glucose levels is difficult or sometimes not possible, so adjacent therapeutic treatments are required. Understanding the molecular pathways underlying the development of DR is key for determining effective therapeutic targets. The pathogenesis of DR is multifactorial and involves a complex network of biochemical and molecular interactions that alter the structural and functional integrity of the retinal vascular environment [5,6,7,8,9,10,11].

Matrix metalloproteinase 9 (MMP9) is a matrix in a group of enzymes belonging to the zinc-metalloproteinase family, which participates in the degradation of the extracellular matrix. Commonly referred to as 92 kDa type IV collagenase, 92 kDa gelatinase, or gelatinase B, this enzyme belongs to the MMP family. MMPs play a central role in the degradation and remodeling of the extracellular matrix and are functionally active in a range of physiological processes, including embryogenesis, tissue repair, angiogenesis, skeletal development, reproduction, cell migration, and cognitive functions such as learning and memory [12,13]. Matrix metalloproteinases (MMPs) are implicated in the regulation of key cellular processes such as programmed cell death and the formation of new blood vessels. Under diabetic conditions, the expression and release of various MMPs are upregulated and have been associated with the development of complications including DR, nephropathy, and cardiomyopathy. Elevated levels of MMP9 and MMP2 have been reported in both the retinal and vitreous tissues of patients with DR, as well as in experimental diabetic models [2,5,14]. Recent research has shown that MMPs have a dual role in the development of DR [2,5,9,15]. In the initial phase of the disease, MMP2 and MMP9 are thought to facilitate the apoptosis of retinal capillary cells, possibly by damaging mitochondria, and they may assist in neovascularization in the next stage [16]. Besides metalloproteinases, a disintegrin and metalloprotease domain (ADAM) is a family of transmembrane proteins that are closely related in terms of their proteolysis and cell adhesion functions, and they have become an interesting target in eye disease research [17,18,19]. ADAM9 possesses enzymatic activity and can associate with multiple cellular elements and extracellular matrix structures. The active metalloproteinase domain of ADAM9 can degrade extracellularly. By degrading matrix components, growth factors, and cytokines, it plays a role in modulating key physiological processes including cell growth, movement, and the formation of new blood vessels [20,21,22]. The current study is focused on comparing the serum ADAM9 and MMP9 levels of patients with DR and healthy individuals, since their effects on cell destruction, proliferation, and many other retinopathies are similar.

## 2. Results

As shown in Table 1, age distributions were comparable across all study groups (control, NPDR, and PDR), with no significant differences detected (*p* = 0.759). Biomicroscopic evaluations of the right and left eyes demonstrated statistically significant intergroup differences (*p* < 0.001 and *p* < 0.001, respectively).

Intraocular pressure in the right eye did not differ significantly between the groups (*p* = 0.242). However, the left eye exhibited significantly higher intraocular pressure in the PDR group compared to both the control (*p* = 0.001) and NPDR groups (*p* = 0.028). (According to the results of post-hoc analysis.)

The visual acuity of both the right and left eyes was significantly lower in the NPDR and PDR groups than in the control group (*p* < 0.001 and *p* < 0.001 for the right eye and *p* < 0.001 and *p* < 0.001 for the left eye, respectively). In addition, visual acuity was lower in the PDR group compared to the NDPR group (*p* < 0.001 for the right eye and *p* < 0.001 for the left eye).

The serum ADAM9 level was significantly lower in the NPDR and PDR groups compared to the control group (*p* = 0.031 and *p* < 0.001, respectively, according to the results of post-hoc analysis). The ADAM9 level was relatively lower in the PDR group compared to the NPDR group, but this difference was not significant (*p* = 0.142, according to the results of post-hoc analysis). The serum MMP9 level was also significantly lower in the PDR group compared to the control and NPDR groups (*p* = 0.039 and *p* = 0.013, respectively, according to the results of post-hoc analysis).

Univariate regression analysis showed that ADAM9 (OR: 0.998; 95% CI: 0.996–0.999; *p* = 0.003) was a potential predictive factor for DR. Multivariate regression analysis also identified ADAM9 (OR: 0.998; 95% CI: 0.997–0.999; *p* = 0.007) as an independent predictive factor for DR (Table 2).

ROC curve analyses were conducted of the intraocular pressure (IOP) in the left eye and ADAM9 in predicting DR.

Both the right and left eyes were initially analyzed (Table 2). However, statistically significant findings were observed only in the parameters for the left eye; thus, further analyses, including multinomial logistic regression and ROC curves, were conducted using data from the left eye (Table 3).

**Figure 1:**
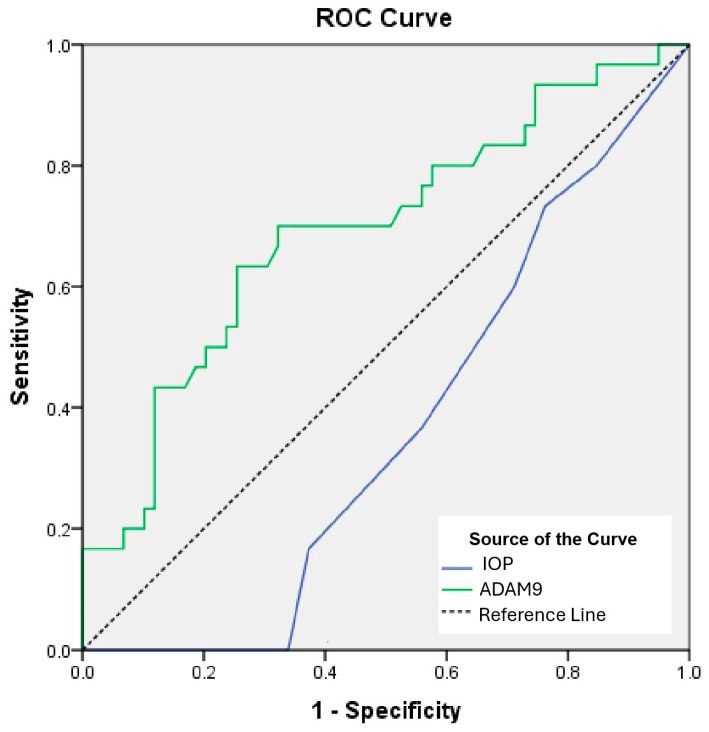
Receiver operating characteristic (ROC) curves. ADAM: a disintegrin and metalloprotease domain, IOP: intraocular pressure.

The ROC curves revealed that the optimal cut-off value of IOP in the left eye was 18.5, with a sensitivity of 33.9% and a specificity of 100% (AUC: 0.362; 95% CI: 0.249–0.476; *p* = 0.035); the optimal cut-off value of ADAM9 was 1194.6 with a sensitivity of 63.3% and a specificity of 74.6% (AUC: 0.693; 95% CI: 0.573–0.812; *p* = 0.003) (Table 3) (Figure 1). While ADAM9 showed significant diagnostic performance (AUC = 0.693), IOP was found unsuitable for clinical use, with an AUC value of 0.362.

## 3. Discussion

DR remains one of the most debilitating chronic complications. However, our knowledge of DR has been greatly enhanced by extensive research into its etiology. The incidence of this disease is increasing, with 190 million people expected to be affected by 2030. Despite extensive research in this field, the precise mechanisms responsible for retinal damage in diabetes and the exact underlying mechanisms still remain elusive [23,24,25,26]. Most individuals with DR are unaware of their condition. Furthermore, DR can be prevented with necessary precautions. Simple tests using human blood samples could help to further elucidate the relationship between disease orientation and retinopathy stages.

This study focused on the identification of important biomarkers for the diagnosis of DR through regression analysis of the ADAM9 marker and sensitivity testing using the left eye ROC curve. As summarized in Table 1, mean ages did not differ significantly among the study groups (control vs. NPDR vs. PDR: *p* = 0.759). However, distinctive variations were noted in the biomicroscopic assessments of both the right and left eyes, indicating significant differences between the groups (*p* < 0.001 and *p* < 0.001, respectively). Our results show that ADAM9 was significantly lower in both DR groups, suggesting that it is a potential marker for detecting retinopathic changes. The serum ADAM9 level demonstrated a significant decrease in both the NPDR and PDR groups when compared to the control group (*p* = 0.031 and *p* < 0.001, respectively). Although the ADAM9 level was relatively lower in the PDR group compared to the NPDR group, this difference did not reach statistical significance (*p* = 0.142). Furthermore, the serum MMP9 level exhibited a significant decrease in the PDR group in comparison to both the control and NPDR groups (*p* = 0.039 and *p* = 0.013, respectively).

In particular, MMP9 levels were significantly lower in the PDR group compared to the control and NPDR groups. As stated in our findings, IOP in the left eye and ADAM9 values may be important biomarkers in the diagnosis and staging of DR. The most striking finding of our study is that when these markers are evaluated together with the ocular values in the left eye, the type and staging of DR can be determined more precisely.

ADAM9 is a protein that has a significant impact in processes such as development, inflammation, degenerative diseases, and cancer through proteolytic processing of cell surface proteins and cell adhesion. Understanding the mechanisms of ADAM9 is critical for the development of therapeutic strategies [27]. Genetic studies on ADAM9 by Liang et al. indicated that it may be a potential therapeutic target for modulating cellular damage in DR [28]. They examined how high-glucose conditions affect the expression of ADAM9 in retinal pigment epithelial cells and showed that ADAM9 expression was markedly increased in a high-glucose environment, and they suggested that this would be associated with an increase in the proteolytic activity of ADAM9, which causes it to cleave its extracellular domain in retinal pigment epithelial cells [28].

Guaiqul et al., in their study investigating the characteristics and potential mechanisms of plus disease in a mouse model of oxygen-induced retinopathy (OIR), showed partial protection against plus disease symptoms in mice lacking the ADAM9 gene after OIR induction [29], suggesting a potential impact of ADAM9 on the development of plus disease. In this context, they showed the importance of understanding the effects of ADAM9 on plus disease in the retina for identifying potential therapeutic targets [29].

On the other hand, ADAM9 and left eye ocular values are important biomarkers for DR and its staging. These markers, when used together with left eye ocular values, may allow a more precise determination of the type and stage of retinopathy.

ROC curve and sensitivity test results also support the efficacy of ADAM9 and left eye ocular markers in the diagnosis of DR and emphasize the potential use of these markers in clinical applications. In conclusion, the findings of our study suggest that ADAM9, MMP9, and left eye ocular parameters may have potential as supportive biomarkers in the evaluation of DR. These findings may help clinicians in the early diagnosis of DR patients and in determining appropriate treatment strategies. In the future, validation of these biomarkers in larger patient groups and support with prospective studies may contribute to the advancement of this field. A limitation of our study is that both eyes could not be modeled simultaneously with correlation-adjusted approaches (e.g., GEE or mixed-effects models) due to the limited sample size; therefore, the advanced analyses were restricted to the eye that demonstrated the most statistically significant findings.

Fabrikantov et al. compared groups with T2DM and NPDR and groups with T2DM and no existing DR or other ocular pathology; they showed a statistically significant increase in MMP9 in the main group of patients compared to the age-controlled group [30].

Gu et al. analyzed 73 gene regions related to DR from 70 published studies in their bioinformatics study. They identified the MMP9 gene as one of the central genes associated with neovascularization by forming a protein–protein interaction network. They stated that MMP9 could be new a target for early neovascularization therapy in the future [31]. Furthermore, MMP-2 is critical for regulating angiogenesis in DR: the fully active form induces endothelial apoptosis. Interestingly, the increase in MMP-9 observed in MMP-2 knockout mice suggests that these two gelatinases can functionally compensate for each other and play synergistic roles in the pathogenesis of DR. Therefore, both enzymes are important for a complete understanding of disease mechanisms [32].

Through endothelial cell and animal studies, Mohommed et al. demonstrated a signaling pathway responsible for H-Ras-mediated activation of MMP9 in the retina, which increases capillary cell apoptosis and participates in the development of DR. They stated that understanding the mechanism behind MMP9 activation may help identify new molecular targets for pharmacological interventions to prevent the onset and progression of DR [33].

Chen et al., in their study on the MMP9 inhibitor minocycline, showed a significant correlation between MMP9 expression and retinal vascular permeability. They concluded that MMP9 is involved in damage to retinal vascular permeability from enhanced glycation end products, and abnormal permeability is partially reversible with minocycline treatment [34].

However, there are limited studies on the subject in the literature. The existing studies also encounter problems in terms of staging, sample diversity, methodological differences, and patient standardization. The precise mechanism by which ADAM9 and MMP9 may contribute to the development of DR is unclear. In conclusion, our findings indicate that serum MMP9 levels differ between stages of DR, with relatively higher values observed in the proliferative stage. While this may reflect a potential association between MMP9 expression and disease progression, the current data do not allow for definitive conclusions regarding its diagnostic or staging utility. Further studies with larger samples and multivariate modeling are needed to clarify the specific role of MMP9 in DR staging.

Although ADAM9 and IOP also showed differences across groups, their diagnostic value appears to be limited based on modest AUC values and sensitivity levels. These parameters may still hold exploratory potential and should be evaluated in larger, prospective studies.

## 4. Materials and Methods

### 4.1. Patients

Patients who were diagnosed with DR and healthy individuals who applied to Amasya University S. S. Training and Research Hospital Department of Ophthalmology were included in the study. They were informed about the study and agreed to be included on a voluntary basis. Age, gender information, and disease anamnesis of the patients were recorded and physical examinations were performed. A detailed eye examination was performed while the patients were admitted to the clinic. A priori power analysis for one-way ANOVA (NPDR vs. PDR vs. control) indicated that 89 participants provided 83.6% power to detect medium-to-large effects (f = 0.35) at α = 0.05. In total, 27 patients with NPDR, 32 patients with PDR, and 30 healthy individuals were included in the study. The diagnosis of DR was made by specialist physicians as a result of routine eye examination, visual acuity (measured using the Snellen chart), biomicroscopy, intraocular pressure measurement, and detailed eye fundus examination after pupil dilation, which were routinely performed in the ophthalmology clinic, and DR patients were included in the DR patient group. Individuals with diseases such as cardiovascular disease, inflammatory disorders, neurodegenerative conditions, or infections were not included in the study.

### 4.2. Collection and Analysis of Blood Samples

Blood samples were routinely requested for serum samples; 5 mL of blood was collected in yellow-capped gel tubes and the samples were slowly inverted 5–6 times. After waiting for at least 30 min, the samples were centrifuged at 1500–2000× rpm for 10 min with a centrifuge device. The serum, which was separated at the top of the tube, was aliquoted and transferred to Eppendorf tubes. The transferred samples were stored in a deep freezer at −80 °C. The parameters and ADAM9 and MMP9 levels were determined using the ELISA (Bioassay Technology Laboratory, Birmingham, UK) method in accordance with the instructions provided with the kit. Serum levels of ADAM9 were measured using commercially available sandwich ELISA kits (Cat. No: E0954Hu) and MMP9 was also quantified using an ELISA kit (Cat. No: E0936Hu), supplied by Bioassay Technology Laboratory (BT Lab, Shanghai, China). The MMP-9 assay has a detection range of 30–9000 ng/L, with a sensitivity of approximately 15 ng/L. The results are given in ng/L. The intra-assay coefficient of variation (CV) was reported to be <10%, and the inter-assay CV was <12% in serum samples. For the ADAM9 assay, the reported intra-assay and inter-assay CVs were typically <8% and <10%, respectively. All measurements were performed in accordance with the manufacturer’s instructions.

### 4.3. Ethical Aspects

This study was conducted in accordance with the Declaration of Helsinki of 1975 (as revised in 2013), and the protocol was reviewed and approved by Amasya University Non-Interventional Clinical Research Ethics Committee on 21 September 2022 (Approval Decision Number: 9/88). All subjects provided written informed consent for inclusion before they participated in the study.

### 4.4. Statistical Analysis

Data analysis was performed using SPSS version 22 (SPSS Inc., Chicago, IL, USA). The suitability of the variables for a normal distribution was checked visually (with histogram and probability graphs) and analytical methods (Kolmogorov–Smirnov/Shapiro–Wilk tests) were used. One-way ANOVA was used to test the differences between groups in terms of normally distributed continuous variables, post hoc least significant difference (LSD) tests were used for pairwise comparisons, and the results were presented as means ± standard deviations (SDs). One-way ANOVA was used to test the differences between groups in terms of normally distributed continuous variables, and Levene’s test was applied to evaluate the homogeneity of variances prior to ANOVA. Post hoc least significant difference (LSD) tests were used for pairwise comparisons, and the results were presented as means ± standard deviations (SDs). Kruskal–Wallis was used to test the differences between groups in terms of continuous variables that were not normally distributed, and Bonferroni correction was applied for post hoc pairwise comparisons. The results were presented as medians and interquartile ranges (IQRs). Qualitative data were compared via the chi-square test and the results are presented as n-percentages (%).

Binary logistic regression analysis was applied to determine whether IOP, ADAM9, and MMP9 were associated with DR. Variables with a *p* value of <0.10 in the univariate analysis were included in the multivariate binary logistic regression model using the Hosmer–Lemeshow test for goodness of fit. The analysis results are presented as odds ratios (ORs) and 95% confidence intervals (CIs). Bonferroni correction was applied to control for multiple testing. Optimal cut-off values of intraocular pressure in the left eye and ADAM9 were determined by receiver operating characteristic (ROC) curve analysis and defined as values that maximize the Youden index (sensitivity + specificity − 1). Alpha values below 0 < 0.05 were used as the statistical significance level.

## 5. Conclusions

In summary, our study underscores the potential of ADAM9, MMP9, and left-eye ocular values as biomarkers for diagnosing and staging DR. When assessed alongside left-eye ocular values, these markers offer increased precision in determining DR type and stage. The results of this study also suggest a specific role of MMP9 in identifying the proliferative stage of DR. Although MMP9 levels differed between stages of DR in our study, these findings should be interpreted with caution. Our current design did not include comprehensive multivariate adjustment for all potential confounders, particularly key systemic factors such as HbA1c and duration of diabetes. Therefore, while MMP9 appears to have stage-related variation, its utility as a staging biomarker should be regarded as exploratory and remains to be confirmed in larger, longitudinal cohorts using adjusted statistical models.

## Figures and Tables

**Table 1:** Data of the groups.

	Control (n = 30)	NPDR (n = 27)	PDR (n = 32)	*p* Value
Age (year)	59.3 ± 1.97	59.96 ± 6.02	59.84 ± 1.65	0.759 ^a^
Biomicroscope—right eye				<0.001 ^b^
Nature	28 (93.33)	23 (85.19)	19 (59.38)	
Cataract	1 (3.33)	2 (7.41)	9 (28.13)	
Pseudophakia	1 (3.33)	2 (7.41)	4 (12.5)	
Biomicroscope—left eye				<0.001 ^b^
Nature	29 (96.66)	25 (92.59)	19 (59.38)	
Cataract	0 (0)	0 (0)	10 (31.25)	
Pseudophakia	1 (3.33)	2 (7.41)	3 (9.38)	
IOP—right eye (mmHg)	16.07 ± 2.12	16.7 ± 2.45	17.28 ± 3.58	0.242 ^a^
IOP—left eye (mmHg)	15.67 ± 1.75	16.63 ± 2.92	18.41 ± 3.97 *^†^	0.002 ^a^
Visual acuity—right eye	1 (0.0)	0.8 (0.6) *	0.4 (0.3) *^†^	< 0.001 ^c^
Visual acuity—left eye	1 (0.0)	0.8 (0.4) *	0.35 (0.3) *^†^	< 0.001 ^c^
ADAM9 (ng/L)	1314.28 ± 481.41	1096.69 ± 329.54 *	951.98 ± 280.11 *	0.001 ^a^
MMP9 (ng/L)	935.04 ± 216.57	965.26 ± 161.74	811.87 ± 287.78 *^†^	0.028 ^a^

^a^ One-way ANOVA. Data are presented as mean ± standard deviation (SD). ^b^ Chi-square test. Data are presented as n and percentages (%). ^c^ Kruskal–Wallis test. Data are presented as median and interquartile range (IQR). * a significant difference at the level of *p* < 0.05 compared to the control group. ^†^ a significant difference at the level of *p* < 0.05 compared to the NPDR group. NPDR: non-proliferative retinopathy patients; PDR: proliferative retinopathy patients; ADAM9: a disintegrin and metalloprotease domain; MMP9: matrix metalloproteinase, IOP: intraocular pressure.

**Table 2:** Univariate and multivariate analyses of predictive factors for DR.

	Univariate			Multivariate		
	OR	95% CI	*p*	OR	95% CI	*p*
IOP—right eye	1.135	0.961–1.341	0.137			
IOP—left eye	1.255	1.053–1.495	0.011	1.237	1.021–1.500	0.03
ADAM9	0.998	0.996–0.999	0.003	0.998	0.997–0.999	0.007
MMP9	0.999	0.997–1.001	0.322			

OR: odds ratio; CI: confidence interval; ADAM9: a disintegrin and metalloprotease domain; MMP9: matrix metalloproteinases, IOP: intraocular pressure.

**Table 3:** ROC curves and prognostic accuracy of IOP in the left eye and ADAM9.

Risk Factor	AUC	95% CI	*p*	Cut-Off	Sensitivity (%)	Specificity (%)
IOP—left eye	0.362	0.249–0.476	0.035	>18.5	33.9	100
ADAM9	0.693	0.573–0.812	0.003	<1194.6	63.3	74.6

AUC: area under the curve; ADAM9: a disintegrin and metalloprotease domain; CI: confidence interval, IOP: intraocular pressure.

## Data Availability

The original contributions presented in this study are included in the article. Further inquiries can be directed to the corresponding author.

## Abbreviations

The following abbreviations are used in this manuscript:

ADAM	A Disintegrin and Metalloprotease
DR	Diabetic Retinopathy
MMP	Matrix Metalloproteinase
IOP	Intraocular pressure

## References

[1] Williams R., Airey M., Baxter H., Forrester J., Kennedy-Martin T., Girach A. (2004). Epidemiology of diabetic retinopathy and macular oedema: A systematic review. Eye.

[2] Kowluru R.A., Zhong Q., Santos J.M. (2012). Matrix metalloproteinases in diabetic retinopathy: Potential role of MMP-9. Expert Opin. Investig. Drugs.

[3] Zhu Z.Y., Meng Y.-A., Yan B., Luo J. (2021). Effect of anti-VEGF treatment on nonperfusion areas in ischemic retinopathy. Int. J. Ophthalmol..

[4] Armulik A., Genove G., Betsholtz C. (2011). Pericytes: Developmental, physiological, and pathological perspectives, problems, and promises. Dev. Cell.

[5] Kowluru R.A., Abbas S.N. (2003). Diabetes-induced mitochondrial dysfunction in the retina. Investig. Opthalmol. Vis. Sci..

[6] Madsen-Bouterse S.A., Zhong Q., Mohammad G., Ho Y.-S., Kowluru R.A. (2010). Oxidative damage of mitochondrial DNA in diabetes and its protection by manganese superoxide dismutase. Free Radic. Res..

[7] Santos J.M., Mohammad G., Zhong Q., Kowluru R.A. (2011). Diabetic retinopathy, superoxide damage and antioxidants. Curr. Pharm. Biotechnol..

[8] Santos J.M., Kowluru R.A. (2011). Role of Mitochondria Biogenesis in the Metabolic Memory Associated with the Continued Progression of Diabetic Retinopathy and Its Regulation by Lipoic Acid. Investig. Opthalmol. Vis. Sci..

[9] Xia H.-Q., Yang J.-R., Zhang K.-X., Dong R.-L., Yuan H., Wang Y.-C., Zhou H., Li X.-M. (2022). Molecules related to diabetic retinopathy in the vitreous and involved pathways. Int. J. Ophthalmol..

[10] Zhang Y., Wang W., Yang A. (2022). The involvement of ACO3 protein in diabetic retinopathy through the PI3k/Akt signaling pathway. Adv. Clin. Exp. Med..

[11] Pieńczykowska K., Bryl A., Mrugacz M. (2025). Link Between Metabolic Syndrome, Inflammation, and Eye Diseases. Int. J. Mol. Sci..

[12] Wang J., Tsirka S.E. (2005). Neuroprotection by inhibition of matrix metalloproteinases in a mouse model of intracerebral haemorrhage. Brain.

[13] Yang Y., Hill J.W., Rosenberg G.A. (2011). Multiple roles of metalloproteinases in neurological disorders. Prog. Mol. Biol. Transl. Sci..

[14] Fridman R., Toth M., Chvyrkova I., Meroueh S.O., Mobashery S. (2003). Cell surface association of matrix metalloproteinase-9 (gelatinase B). Cancer Metastasis Rev..

[15] Mishra M., Kowluru R.A. (2017). Role of PARP-1 as a novel transcriptional regulator of MMP-9 in diabetic retinopathy. Biochim. Et Biophys. Acta Mol. Basis Dis..

[16] Klein T., Bischoff R. (2010). Physiology and pathophysiology of matrix metalloproteases. Amino Acids.

[17] Wolfsberg T.G., Primakoff P., Myles D.G., White J.M. (1995). ADAM, a novel family of membrane proteins containing A Disintegrin And Metalloprotease domain: Multipotential functions in cell-cell and cell-matrix interactions. J. Cell Biol..

[18] Li C.R. (2023). Role of lymphotoxin alpha as a new molecular biomarker in revolutionizing tear diagnostic testing for dry eye disease. Int. J. Ophthalmol..

[19] Masli S., Akpek E.K. (2022). Reduced tear thrombospondin-1/matrix metalloproteinase-9 ratio can aid in detecting Sjogren’s syndrome etiology in patients with dry eye. Clin. Transl. Sci..

[20] Haoyuan M., Yanshu L. (2020). Regulatory factors and cancer-related physiological effects of ADAM9. Cell Adhes. Migr..

[21] Cauwe B., Van den Steen P.E., Opdenakker G. (2007). The Biochemical, Biological, and Pathological Kaleidoscope of Cell Surface Substrates Processed by Matrix Metalloproteinases. Crit. Rev. Biochem. Mol. Biol..

[22] Overall C.M., Kleifeld O. (2006). Tumour microenvironment—Opinion: Validating matrix metalloproteinases as drug targets and anti-targets for cancer therapy. Nat. Rev. Cancer.

[23] Drankowska J., Kos M., Kościuk A., Marzęda P., Boguszewska-Czubara A., Tylus M., Święch-Zubilewicz A. (2019). MMP targeting in the battle for vision: Recent developments and future prospects in the treatment of diabetic retinopathy. Life Sci..

[24] Zheng Y., He M., Congdon N. (2012). The worldwide epidemic of diabetic retinopathy. Indian. J. Ophthalmol..

[25] Kour V., Swain J., Singh J., Singh H., Kour H. (2024). A Review on Diabetic Retinopathy. Curr. Diabetes Rev..

[26] Mounirou B.A.M., Adam N.D., Yakoura A.K., Aminou M.S., Liu Y.T., Tan L.Y. (2022). Diabetic Retinopathy: An Overview of Treatments. Indian J. Endocrinol. Metab..

[27] Chou C.W., Huang Y.K., Kuo T.T., Liu J.P., Sher Y.P. (2020). An Overview of ADAM9: Structure, Activation, and Regulation in Human Diseases. Int. J. Mol. Sci..

[28] Liang Z., Lu C., Feng T., Gao X., Tu Y., Yang W., Wang Y. (2022). Circ-ADAM9 Promotes High Glucose-Induced Retinal Pigment Epithelial Cell Injury in DR via Regulating miR-338-3p/CARM1 Axis. J. Ophthalmol..

[29] Guaiquil V.H., Hewing N.J., Chiang M.F., Rosenblatt M.I., Chan R.V.P., Blobel C.P. (2013). A murine model for retinopathy of prematurity identifies endothelial cell proliferation as a potential mechanism for plus disease. Investig. Opthalmol. Vis. Sci..

[30] Fabrikantov O.L., Lev I.V., Agarkov N.M., Osmanov R.E. (2022). Prognostic value of matrix metalloproteinases in the development of diabetic retinopathy in the elderly. Adv. Gerontol..

[31] Gu C., Lhamo T., Zou C., Zhou C., Su T., Draga D., Luo D., Zheng Z., Yin L., Qiu Q. (2020). Comprehensive analysis of angiogenesis-related genes and pathways in early diabetic retinopathy. BMC Med. Genom..

[32] Esparza J., Kruse M., Lee J., Michaud M., Madri J.A. (2004). MMP-2 null mice exhibit an early onset and severe experimental autoimmune encephalomyelitis due to an increase in MMP-9 expression and activity. FASEB J..

[33] Mohammad G., Kowluru R.A. (2011). Diabetic retinopathy and signaling mechanism for activation of matrix metalloproteinase-9. J. Cell. Physiol..

[34] Chen Y.D., Xu X., Xia X., Wu H., Liu K., Zheng Z., Zhu D. (2008). MMP9 Is Involved in Glycation End-Products Induced Increase of Retinal Vascular Permeability in Rats and the Therapeutic Effect of Minocycline. Curr. Eye Res..

